# Lectures on the Processes of Repair and Reproduction after Injuries

**Published:** 1849-11

**Authors:** James Paget

**Affiliations:** Professor of Anatomy and Surgery to the College


### RECORD OF MEDICAL SCIENCE.

### ANATOMY AND PHYSIOLOGY.

*Lectures on the processes of Repair and Reproduction after Injuries. Delivered at the Royal College of Surgeons of England.* By James Paget, F. R. C. S., Professor of Anatomy and Surgery to the College.

### LECTURE I.—CONCLUDED.

The capacity of repairing damages sustained by injury, and to reproduce lost parts, appears to belong, in some measure, to all bodies having definite form and construction. It is not an exclusive property of living beings; for even crystals will repair themselves when, after pieces have been broken from them, they are placed in the same conditions in which they were first formed. I know not what amount of mutual illustration, if any, the repair of crystals and living bodies may afford; but, in any case, we may trace here something like an universal property of bodies that are naturally and orderly constructed; all, in favorable circumstances, can repair at least some of the damages to which they are liable from the violence of external forces. The power of reproduction is distributed in various degrees through the animal kingdom. The general statement sometimes made, that the reparative power in each species bears an inverse ratio to its position in the scale of animal life, is certainly not proved; and many instances are contrary to it, such as the great reparative power possessed by the lizards, and the apparently complete absence of it in the perfect insects. That which is generally true is, that the reparative power bears an inverse proportion to the amount of germ power consumed in the developement and growth of the individual, and in its maintenance in the perfect state. There are facts enough to prove that the power which can issue from the mysterious properties of a germ is limited; that it is not ever communicated to an indefinite quantity of matter; and there are enough to justify the hypothesis, that the germ power, thus limited, is in some measure consumed in the developement of every new structure, and, in a less measure, in the growth and maintenance of those already formed.

First, then, it appears constantly true, that the reparative power is greater in all parts of the young than of the older of all species. Even when we compare individuals that have all attained their highest development and growth, this rule seems to be true. We know it from general observations of the results of similar injuries, and in persons of different ages: numerous as the exceptions may be, the general rule seems true. And it is yet more eminently true in the case of some lower animals. It seems certain that the capacity for the repair or reproduction of injured parts is much more diminished by development than by growth or maintenance of the body; in other words, to improve a part requires much more, and more perfect, formative power, than to increase it does.

If we may thus interpret the facts, they will be collateral evidence for the belief, that, in different species of animals, the reparative power will bear an inverse ratio to the amount of development already passed through; so that, for each species, in its perfect state, the reparative power might be measured by the degree of likeness between the embryonic and the perfect form, structure, and composition. The greater the sum of dissimilarities in all these respects between the embryo and the perfect animal, the less seems to be the reparative power in the latter. It is consistent with this that the highest amount of reparative power exists in these lowest polypes, in which the materials of the germ-mass yet remain, not transformed, but multiplied, and, as it were, grouped into the shape of their bodies. In the fresh water Hydra, the power of the germ appears communicated without loss to all the cells that descend by multiplication from the original germ-cells, provided only the cells are not developed into higher forms or into subserviency to special functions. In the Hydra viridis and Hydra fusca it seems literally true that any minute cluster of cells, derived by mere multiplication from those of the germ-mass, may, after being separated from the perfect body, reproduce the perfect form. This is the result of the numerous experiments performed on the Hydra by Trembley. He cut an Hydra into four pieces: each became a perfect Hydra; and while they were growing, he cut each of these four into two or three. These fractions of the quarters being on their way to become perfect, he again divided these, and thus he went on, till from the one Hydra he obtained fifty. All these became perfect; he kept many of them for more than two years, and they multiplied by their natural gemmation just as much as others that had never been divided. So that a division by fifty did not perceptibly diminish the power of the germ present in the polype that was thus subdivided. Again, he cut similar polypes longitudinally, and in an hour or less each half had rolled itself up, and seamed up its cut edges, so as to be a perfect Hydra. He split them into four,—he quartered them,—he cut them into as many pieces as he could, and nearly every piece became a perfect Hydra. He slit one into seven pieces, leaving them all connected by the tail, and the Hydra became seven-headed, and he saw all the heads eating at the same time. He cut off the seven heads, and hydra-like, they sprang forth again. But the heads of Lennæan Hydræ perished after excision: the heads of this Hydra grew for themselves bodies, and multiplied with as much vigor as their parent trunk. So in the Actiniæ, which, after bisection, form two perfect individuals,—and the Holothuriæ, which, as Sir J. G. Dalyell has observed, when touched will sometimes eject all their viscera, and yet in three or four months will have all their viscera reproduced. And so of the Anellata, the young Nereids, and species of Nais, on which Spallanzani and others made their experiments. I need not say how consistent all this is with the doctrine of parthenogenesis which my colleague has been explaining, where creatures are produced without previous impregnation of the parent. But I shall now notice some experiments made by Sir J. G. Dalyell, which carry us further in illustration,—facts which seem to show in these lower animals such a gradual approach to perfection as appears in the higher. In the reproduction of the Hydra Tuba he has found, that when cut in halves, each half may regain the perfect form; but this perfect form is regained only very slowly, and, as it were, by a gradual improvement of parts that are, at first, ill-formed.

Again, in the Tubularia indivisa, a specimen was cut near its root, and, after the natural fall of its head, the summit of its stem was cloven. An imperfect head was first produced, at right angles to the stem, from one portion of the cleft; after its fall, another and more nearly perfect one was regenerated, and, as it grew, improved yet more. A third appeared, and yet a fourth, which was yet more nearly perfect, though the stem was thick, and the tentacula imperfect. The cleft was almost healed; and now a fifth head was formed quite perfect; and after it, as perfectly, a sixth and seventh head.

The lower half of this specimen had been cut off four months after the separation of the stem. Its upper end bore—first, an abortive head; then, secondly, one which advanced further in development; a third, much better; and then, in succession, other four, which were all well formed.

The upper portion of this lower half of the stem now showing signs of decay, a portion was cut from its lower part, and further manifested the reproductive power of the stem; for three heads were produced from the upper end of the piece cut off, and four from the lower end of the upper piece which had seemed to be decaying. In 550 days this specimen had grown 22 heads.

Now, I cannot but think, that we have here the very type of what we have to watch in various diseases of the human frame, in which each of the replacing parts becomes more and more near to the character of the perfect individual.

The power of reconstructing a whole and perfect body, by the development of a fragment, is probably limited to the species that can propagate by spontaneous fission or gemmation, or that increase their size, as some of the Anellata do, by the successive addition of rings that are developed after the manner of gemmules from those that precede them. Where this power is not possessed, there, whatever be the position of the species in the animal scale, the reparative power appears to be limited to the reproduction of lost members,—as legs, claws, a part of the body, the head, an eye, the tail, and the like; yet, within this limit, the rule seems to hold good,—that the amount of reparative power is in an inverse ratio to that of the developement through which the animal has passed in its way to the attainment of perfection. The experiments of Mr. Newport show, that, among the insects, the reparative power, in the complete state, is limited to the orders in which that state is attained by a comparatively simple and direct course of development, as the Myriapoda and Phasmidæ, and some of the Orthoptera. These can reproduce their antennæ, and their legs, after removal or mutilation; but their power of reproduction diminishes as their developement increases, as in the Myriapoda, where such reparative power apparently ceases, when, after the last casting of their integuments, their development is completed.

In the higher hexapod insects, such an amount of reproductive power has been seen in only the larval state,—none of them, in its perfect state, can reproduce an antenna, or any other member. This is the more remarkable, when the higher insects are compared with the Arachnida; and yet stronger when we compare the higher insects and the several species of salamander, in which so profuse a reproduction of the limbs has been observed. Many other instances appear to support the rule, that the reparative power in each perfect species is in an inverse proportion to the amount of change through which it has passed in its development from the embryonic to the perfect state, and that the power is the same by which perfection is first achieved, and by which, when lost, it is recovered.

This is, again, generally confirmed in the Vertebrata; but as I shall have to speak of this in future lectures, I will only say that, in the highest Vertebrata, and in man, a true reproduction after loss or injury, seems limited to three classes of parts:—

1. To those which are formed entirely by nutritive repetition.

2. To those which are of lowest organization, and of lowest chemical character.

3. To those which are inserted in other tissues as accessories, as connecting or incorporating them with the other structures of vegetative or animal life.

With these exceptions, injuries or losses in the human body are capable of no more than repair, in its more limited sense.

There are certain considerations which may excuse these references to facts in physiology in which, as surgeons, we may feel but little interest. If ever we are to escape from the obscurities and uncertainties of our art, it must be through the study of those highest laws of our science, which are expressed in the simplest terms in the lives of the lowest orders of creation. It was in the search after the mysteries of generation, that the first glance was gained of the largest truth in physiology—the truth of the development of ova through partition and multiplication of the embryo-cells. So may the study of the repair of injuries sustained by the lowest polypes lead us to the clearer knowledge of that law, in reliance upon which alone we dare to practice our Profession. Already we seem to have had the foreshadowings of the law through which the discovery may be made; and in watching these processes we only see the progress of the yet larger law which expresses our Maker's will for the recovery of all lost perfection. To this train of thought we are guided by the remembrance, that the healing of the body was ever chosen as the fittest emblem of his work whose true mission was to raise man's fallen spirit and repair the injuries he had sustained; and that once, the healing power was exerted in a manner purposely so confined as to advance, like that which we can trace, by progressive stages, to the complete cure. For there was one, upon whom, when the light of Heaven first fell, so imperfect was his vision, that he saw, confusedly, "men, as trees walking;" and then, by a second touch of the Divine hand, was "restored, and saw every man clearly." Thus guided by the brighter light of revelation, it may be our privilege, while we study the things of which I have been speaking, to gain, by the illustrations of analogy, a clearer insight into the oneness of the plan by which things spiritual and corporeal are directed. Even now, we may trace some analogy between the acts of the body and those of man's intellectual and moral nature. As in the development of the germ, so in the history of the human spirit, we may discern a striving after perfection—after a perfection, not viewed in any present model (for the human model was marred almost as soon as it was formed,) but manifested to the enlightened reason in the "express image" of the "Father of Spirits." And so, whenever, through human frailty, amid the violences of the world, and the remaining infection of our nature, the spirit loses aught of the perfection to which it was once admitted, still its implanted power is ever urgent to repair the loss. The same power, derived and still renewed from the same parent, working by the same appointed means, and to the same end, restores the fallen spirit to nearly the same perfection that it had before. Then, not unscarred, yet living—the spirit still feels its capacity for a higher life, and presses to its immortal destiny. Here the analogy ceases. We may watch the germ-power developing the body into all its marvellous perfection and exact fitness for the purpose of its existence in the world; but this purpose accomplished, it passes its meridian, and then we trace it through the gradual decay of life and death. But, for the human spirit that has passed the ordeal of the world, there is no such end. Emerging from its imprisonment in the body, it soars into the element of its higher life; there, in unchanging youth, its powers expand, as the vision of the Infinite unfolds before it; there, in the very presence of its Model, its Parent, and the Source of all its power, it is "like Him, for it sees him as He is."—*Med. Times.*

